# Multiple Epidemic Wave Model of the COVID-19 Pandemic: Modeling Study

**DOI:** 10.2196/20912

**Published:** 2020-07-30

**Authors:** Efthimios Kaxiras, Georgios Neofotistos

**Affiliations:** 1 Department of Physics Harvard University Cambridge, MA United States; 2 Institute of Advanced Computational Science J.A. Paulson School of Engineering and Applied Sciences Harvard University Cambridge, MA United States

**Keywords:** COVID-19, multiple waves, transmission, intervention measures, simulations, modeling, pandemic response index, pandemic, virus, intervention

## Abstract

**Background:**

Intervention measures have been implemented around the world to mitigate the spread of the coronavirus disease (COVID-19) pandemic. Understanding the dynamics of the disease spread and the effectiveness of the interventions is essential in predicting its future evolution.

**Objective:**

The aim of this study is to simulate the effect of different social distancing interventions and investigate whether their timing and stringency can lead to multiple waves (subepidemics), which can provide a better fit to the wavy behavior observed in the infected population curve in the majority of countries.

**Methods:**

We have designed and run agent-based simulations and a multiple wave model to fit the infected population data for many countries. We have also developed a novel Pandemic Response Index to provide a quantitative and objective way of ranking countries according to their COVID-19 response performance.

**Results:**

We have analyzed data from 18 countries based on the multiple wave (subepidemics) hypothesis and present the relevant parameters. Multiple waves have been identified and were found to describe the data better. The effectiveness of intervention measures can be inferred by the peak intensities of the waves. Countries imposing fast and stringent interventions exhibit multiple waves with declining peak intensities. This result strongly corroborated with agent-based simulations outcomes. We also provided an estimate of how much lower the number of infections could have been if early and strict intervention measures had been taken to stop the spread at the first wave, as actually happened for a handful of countries. A novel index, the Pandemic Response Index, was constructed, and based on the model’s results, an index value was assigned to each country, quantifying in an objective manner the country’s response to the pandemic.

**Conclusions:**

Our results support the hypothesis that the COVID-19 pandemic can be successfully modeled as a series of epidemic waves (subepidemics) and that it is possible to infer to what extent the imposition of early intervention measures can slow the spread of the disease.

## Introduction

The coronavirus disease (COVID-19) pandemic has produced a great number of studies that aim to understand the dynamics of the disease spread and predict its future evolution (see, for example, [1-8]). Different types of models can be assumed to describe this dynamic evolution. In studying past epidemics, scientists have systematically applied “random mixing” models that assume that an infectious individual may spread the disease to any susceptible member of the population, as originally proposed by Kermack and McKendrick [9]. Recent approaches consider mobility and contact networks [10,11], epidemic waves attributable to community networks [12], subepidemic modeling [13], Bayesian modeling and inference [14], models of spatial contacts in large-scale artificial cities [15], and power-law models of infectious disease spread [16], to name but a few representative examples.

At present, there is a trove of data from different countries that can serve to put strict limits on plausible models. In this paper we use simulations from agent-based models and simple analytical solutions to fit reported data from a range of countries. This provides a comprehensive picture of likely scenarios of how the disease evolved in various countries. These scenarios can be useful in predicting the future spread of the disease and provide insight on how the imposition of social-distancing measures can be effective in containing or slowing its spread.

Our work is based on two premises. First, the apparent regular features in the reported infections in many countries are not random but rather contain useful information, as their persistence and regularity suggest. Second, there is a general underlying dynamic of the spread of the disease, *in spirit* similar to the original Kermack-McKendrick model of three populations [9], the “susceptible population” *S*(*t*), the “infected/infectious population” *I*(*t*), and the “removed/recovered population” *R*(*t*), which are related by *S*(*t*) + *I*(*t*) + *R*(*t*) = *N*, where *N* is the total population. The time evolution of the *susceptible-infectious-removed* (SIR) populations is described by the equations:

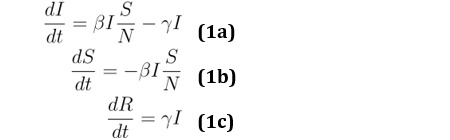



The SIR model involves two positive parameters, *β* and *γ*, which have the following meanings:

- *β* describes the effective contact rate of the disease; an infected individual comes into contact with *β* other individuals per unit time (the fraction that are susceptible to contracting the disease is *S/N*).
- *γ* is the mean removal (recovery) rate, that is, 1*/γ* is the mean period of time during which an infected individual can pass it on before being removed from the group of the infected individuals.

However, the time evolution of the SIR populations, as captured by the linear first-order differential equations of the Kermack-McKendrick model, produce behavior that is much simpler than the actual reported data of infections. Therefore, more detailed (microscopic) models of how the disease is spread from one infected individual to others are required to produce features that can emulate real data. In this study, we consider the simplest possible microscopic model to motivate the reasons that underlie the common features of real data. We then use these results to propose a simple analytical model for fitting the data with a few parameters. Finally, we use the results of the fitting to draw some insights on the actual evolution of the disease in representative countries.

## Methods

### The Microscopic Agent-Based Model

**Figure 1:**
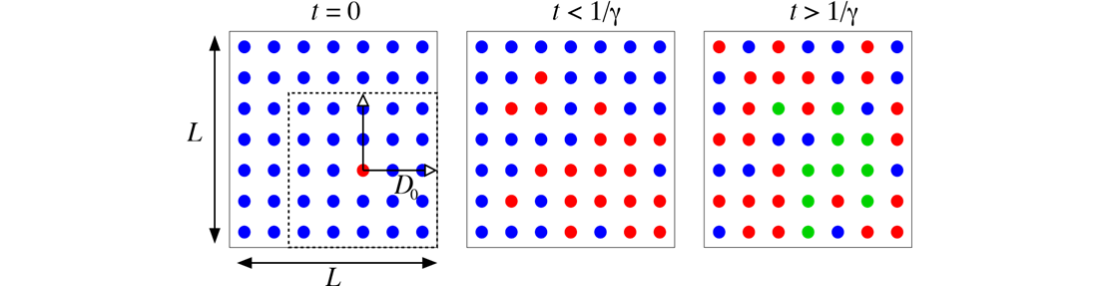
Illustration of the agent-based simulation model on a 2D grid of size *L* × *L*; in this illustration *L*=7, while in our simulations we considered *L*=1000. At *t*=0 (left panel) all the population is susceptible (blue dots), except for one infected individual (red dot). For a period *t* < 1/γ (middle panel) the original infected individual can infect others in a neighborhood within a range ±*D*_0_ in each direction (shown by the dashed square), and each of those infected individuals also infects others within a corresponding range. For *t* > 1/γ (right panel) some of the infected individuals have recovered (green dots), depending on when each was infected.

To understand the dynamics of the epidemic in more detail, we use a more detailed model based on individual agents, which are also classified as susceptible *S*, infected/infectious *I*, and removed/recovered *R*, that exist on a 2D regular grid of points. Each of these agents starts as susceptible and can be infected by another infectious with probability *β* per unit time (which we take here to be 1 day), and once infected can infect other individuals within a range ±*D*_0_, as illustrated in Figure 1.

The size of this range turns out to be a crucial quantity, justifying the quest for social distancing measures to contain or slow down the spread of the disease. After being infected, an agent remains infectious for a period of 1*/γ* days, at which point the agent is removed (recovered) from the infectious population and can neither be infected again (has acquired immunity) or infect others. We suggest that the “microscopic” model is likely to be closer to the actual evolution of the disease than the continuous populations model represented by equation (1). The size of the 2D model, a square of length *L* in the example discussed below, corresponds to a small, uniformly populated “virtual country” of population *N* = *L* × *L*.

**Figure 2:**
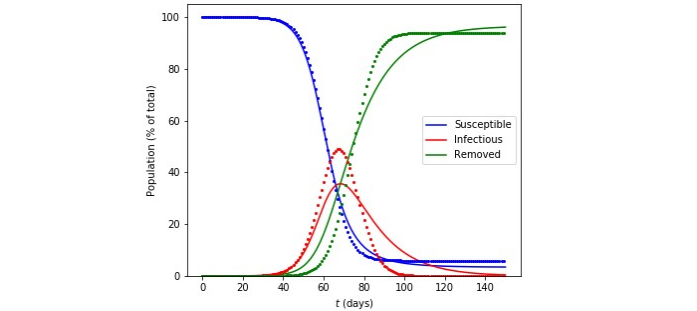
Comparison of the numerical solution to the susceptible-infectious-removed equations (solid lines) and agent-based simulations (points) for a population N. In both cases, we use N=10^6^, β=.25 per day, and 1/γ = 14 days. For the simulations, the range parameter is *D*_0_=50 (see text for details).

We first provided a comparison of the numerical solution of the continuous SIR populations, represented by equation (1) and the agent-based simulations. We chose a total population of *N*=10^6^ for both cases and a number of *I*(0)=4 infected agents, distributed randomly on the 2D grid in the case of the agent-based simulations. In Figure 2, we give a comparison of the evolution of the three populations as a function of time for a total period of 150 days, by which time the infectious population has been reduced to zero and the susceptible and removed populations have reached their long-term asymptotic values in both models. Although the overall behavior of the three populations in the two models is similar, the tail of the *I*(*t*) population is quite “fatter” for the solution of the differential equations. The behavior of the tail is important, as it determines the rate at which the total number of infections (cumulative) grows with time, a subject of active research [17]. The value of the range *D*_0_ in the simulations can be chosen at will up to *D*_0_=*L/*2. The continuous SIR model *contains no information* on this range, which must be somehow included in the effective value of *β*. Keeping the value of *β* the same and adjusting the range *D*_0_ and the initial condition for *I*(0) in the continuous model, we can obtain reasonable agreement between the two models, as shown by an example in Figure 1. In this example, the evolution of the *S*(*t*) population is captured well through the entire time range, except for the asymptotic value. This value is important, because it corresponds to the portion of the population that has not been infected at the end of the epidemic and is given by *N* − *R*_tot_, where *R*_tot_ is the total number of removed; this is also equal to the time integral of the infected population divided by the mean period of infection, 1*/γ*, as can be easily derived from equation (1c):

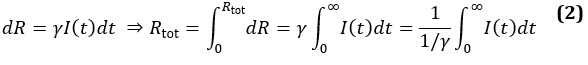



**Figure 3:**
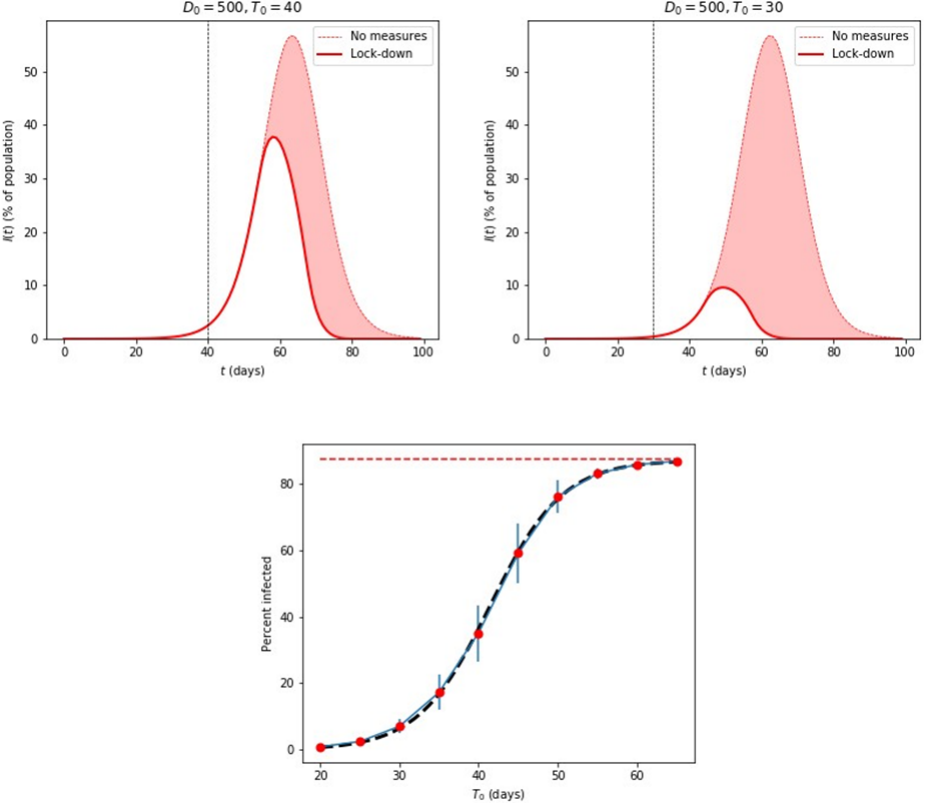
Top: Epidemic disease simulations of the infected population, *I*(*t*), as a percentage of the total population, using 1 million individual agents on a 2D grid, with different lockdown dates, *T*_0_, after the initial cases at *t*=0. The shaded curve in the background corresponds to *I*(*t*) with no lockdown measures. For these simulations, β=.25, 1/γ = 14 days, and λ=2.5 days. Bottom: The percent of the total infected population, *R*_tot_(*T*_0_) for different values of *T*_0_, ranging from 0.93%, for *T*_0_=20 days, to 86.7%, for *T*_0_=65 days. The error bars represent standard deviations from averaging over 30 samples in each case (for the largest and smallest values, the error bars are too small to be visible). The black dashed curve is the fit from equation (4). The red dashed line represents the value for no lockdown measures, which is 87.4%.

An important consideration in the dynamics of the disease is the effect of measures that restrict the movement of individuals in a population. This can easily be captured in the agent-based simulation model by taking a time-dependent value for the range that each infected individual has, namely:

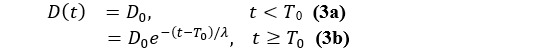



where *T*_0_ is the time at which the measures are imposed; both *t* and *T*_0_ are measured from the time of the first infections, defined as *t*=0. Since *D*(*t*) → 0 for *t* →∞ (assuming *λ*>0) the behavior of the range corresponds to “lock-down” measures in which individuals are restricted to a small range and eventually cannot infect anyone else (they are in “quarantine”). We use *λ*=2*.*5 days in our simulations, which means that from the moment that the measures are imposed (*t*=*T*_0_), the initial range is reduced by a factor of ∼20 for each week that passes. The effect of lockdown measures is quite dramatic, as shown in Figure 3. To provide a quantitative measure of this effect, we first let *D*_0_ be the largest possible, *D*_0_ = *L/*2 (half the size of the grid on which the agents live) and then consider several values of *T*_0_, from 20 to 65, the last value being the time where the maximum of *I*(*t*) occurs in the case of no imposition of restrictions, such as lockdown. A useful measure to quantify this effect is the total population of infected individuals over the course of the epidemic scaled by the mean period of infection 1*/γ*, which is the same number as the total population recovered, see equation (2). This quantity, given as a percent of the total population, is shown in Figure 3 for the whole range of *T*_0_ values we considered.

The asymptotic value, reached for *T*_0_=65 is 86.7% (±0*.*33%), was almost equal to the value when no lockdown measures were imposed, 87.4% (±0*.*06%); this last value corresponds to the “herd immunity” limit for the parameters we have used in the present simulation. The smaller *T*_0_, the lower *R*_tot_ is, reaching the value of 0.93% (±0*.*34%) for *T*_0_=20. The behavior of these values is well approximated by the expression:

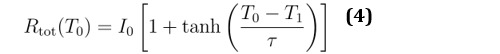



with *I*_0_=43*.*7, *τ*=9*.*1 days, and *T*_1_=41*.*5 days. A clear conclusion from this set of results is that the early imposition of measures makes a significant difference in the total infected population; for example, in a country with a total population of 10 million, the imposition of measures 20 days after the first few reported cases can reduce the total number of infected from 8.74 million to 93,000. The assumptions in this example involve allowing free movement of all persons for the entire period of the disease in the worst-case scenario to full quarantine within 2 weeks after imposing lockdown measures, which reduces the initial range spanning the entire country by a factor of 270, enough to essentially stop any disease transmission.

**Figure 4:**
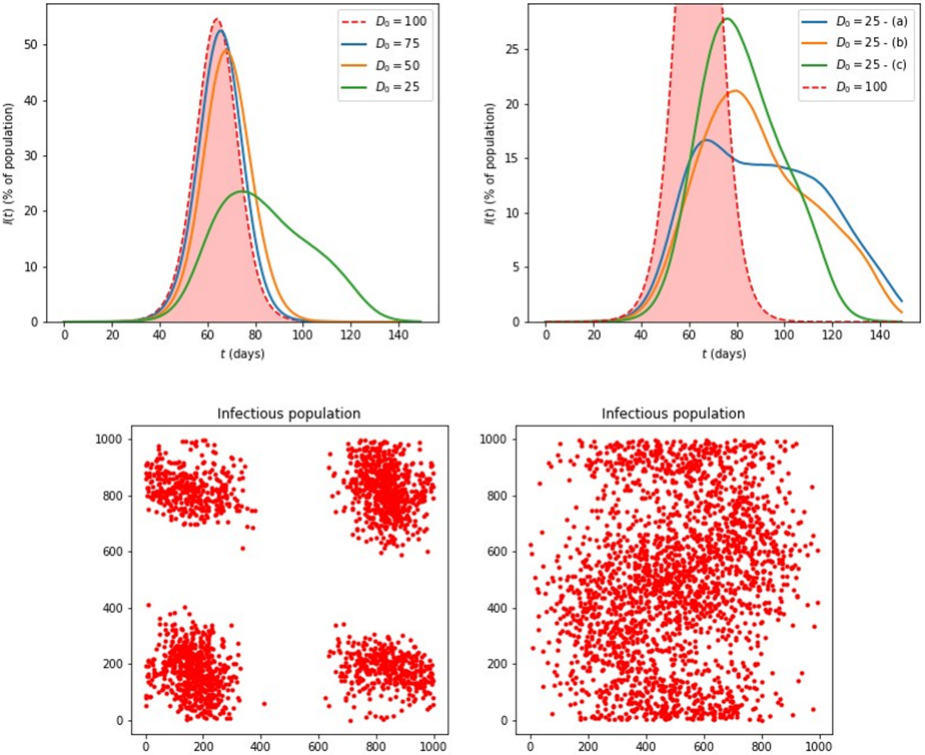
Top row: Simulations based on individual agents with a finite range of spreading the disease, *D*_0_. Left: various values of *D*_0_; the case *D*_0_=100 already approaches the limit of free motion throughout the system, *D*_0_=L/2, see also Figure 3. Right: several instances of *D*_0_=25, labeled (a), (b), (c), exhibiting multiple bumps and long tails. Bottom row: the actual infected populations at the same time moment *t*=30 days, for *D*_0_=50 (left) and *D*_0_=100 (right).

An interesting exercise is to consider what are the effects of *finite D*_0_ much smaller than the size of the system (our “virtual country”). We give some examples of such simulations in Figure 4. For *D*_0_=100 or larger, the result is essentially the same as that of the limiting case of free motion throughout the entire system, which was discussed in Figure 3 (background curves with no lockdown measures). For *D*_0_≤75, the curves start deviating from this behavior and even exhibit more interesting behavior, with additional “bumps” in the descending part and long tails, as the case *D*_0_=25 shows. In fact, for *D*_0_=25 the curves are not unique and depend on the initial random distribution of the infected individuals at *t*=0; we give an example in Figure 4. Within our simple microscopic model, this behavior arising from small values of *D*_0_ (like the case *D*_0_=50 in Figure 4) can be easily explained; it corresponds to several small clusters of infections, which spread through the country in waves, as one cluster eventually becomes all removed, but before reaching this point, some infected individuals have moved to a region where there were no infections at all, starting a second wave of infections. We emphasize that, independent of the initial conditions that determine the number and time of the subsequent waves’ occurrence, for a given value of small *D*_0_, the asymptotic value of *R*_tot_ for *t*→∞ is always the same and of course depends on the specific value of *D*_0_. This simply means that the total number of infections is determined by the effective range of interactions and, given enough time, the disease will infect the same total number of agents no matter how it proceeds from one cluster to others. This behavior of disease evolution may be actually close to what is seen in reported data, as described in the following section.

In contrast to the situation for small values of *D*_0_, for larger values of *D*_0_ all the curves for *I*(*t*) are identical and the behavior is that of one wave smoothly spreading through the entire system, as shown also by an example (*D*_0_=100) in Figure 4. The critical value separating these two regimes is between 100 and 75 in our simulations. It would be interesting to study what this critical value is and what is its relation to the system size *L* in a more systematic manner using methods from statistical physics.

### The Multiple Wave Forced-SIR Model

Recently, the forced-SIR (FSIR) model was proposed by the authors and used to describe the evolution of the COVID-19 pandemic in a representative set of countries [18]. This model treated the evolution of the infected population as a single wave (single peak wave). It contains three adjustable parameters that are estimated for each country by fitting actual data. However, the single-wave assumption cannot explain the entire incidence curve (infected population curve) in each country. Wavy patterns (“bumps”) are evident in the actual data for many countries, which cannot be attributed to simply random fluctuations, due to their regularity and similarity among several countries in which, at first glance, the disease was at different levels of severity. Here, we expand the analytical FSIR model to capture the multiple waves (subepidemics), underlying a country’s overall incidence curve. This is akin to the case of finite *D*_0_ discussed previously in the agent-based model. We applied this multiple wave analytical model to a representative set of 18 countries, in all of which the behavior of the infection as a function of time is accurately represented by our model.

In its original version, FSIR applies to a single epidemic wave, in which the infected population is given by the expression:

*I*(*t*) = *N* − *S*(*t*) − *R*(*t*) **(5)**

with the approximate solution given by:

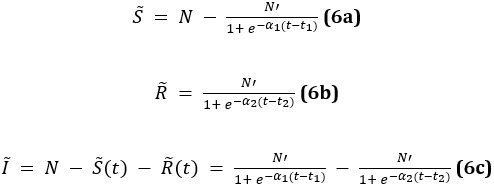



where *N*′, *α*_1_*, α*_2_, *t*_1_*, t*_2_ are treated as adjustable parameters, with *t*_1_ and *t*_2_ representing the times at which the 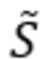 and 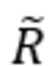 populations reach their sigmoid midpoint values, respectively. Here, we extend this model to allow for multiple waves that capture the subepidemics in the infected population of a country. As argued in the agent-based microscopic model previously presented, several clusters of infections can appear in a country in waves; each cluster eventually becomes all removed, but before reaching this point, some infected individual has moved to a region where there were no infections at all, starting a second local wave of infections. In the extended model, we assume each wave is captured by a function described by equation (6), with different values of the parameters involved.

We apply this extended model to fit the multiple wave behavior of infected populations in different countries, as obtained from the European Centre for Disease Prevention and Control [19], for a period ending on May 16, 2020, which corresponds to 120 days from the onset of the exponential growth of reported cases in China. To obtain a meaningful fit, we had to consider data for each country that showed a monotonic increase at the beginning. This means that a few data points in each case were excluded, as they corresponded to sporadic reports of a few isolated cases, typically 1-10 in a given day, interspersed by several days of zero cases. In practice this means that the fitting begins at a certain cutoff day denoted as *t*_0_.

As in the case of the original FSIR model, to make the fit more robust and simpler, we chose the *α*_1_ and *α*_2_ parameters to have the same value *α*_1_=*α*_2_=*α*=0*.*25. We have found this to be the optimal value for the countries we considered. Moreover, a common value for the exponential decrease of the susceptible population, which is captured by the value of *α*_1_, and for the exponential increase of the removed population, which is captured by the value of *α*_2_, is actually more consistent with the agent-based simulations, as described in Section II.

Finally, instead of using *t*_1_ and *t*_2_ for each wave as independent parameters, we elected to use as independent parameters *t*_1_ and ∆*t* = *t*_2_ − *t*_1_. To make the multiple-wave fit more robust, simpler, and systematic, we chose ∆*t* =14 (days) for all waves, which is a reasonable choice, as it corresponds to a common mean time period of 14 days before the infected individual is removed (∆*t* = 1/*γ* in the agent-based microscopic model). This mean time-period has been imposed as a quarantine measure for the majority of countries imposing measures (interventions) and is consistent with a reported estimated median time of approximately 2 weeks from onset to clinical recovery for mild cases [20]. This condition leaves two adjustable parameters per subepidemic that can be varied to obtain the best fit to the data, namely the onset time *t*_1_, which corresponds to the midpoint of the sigmoid representing the decline of the susceptible population, and *N*′, which is a parameter representative of the number of daily cases near the peak of the infected population curve in the given wave. *N_T_*, the total number of infected in the given wave, can readily be obtained. The best fit here is defined in the Root-Mean-Square (RMS) sense. The model parameters were determined by employing the Levenberg-Marquardt algorithm.

## Results

### Application to Representative Countries

We were able to obtain reasonable fits for over 30 countries from the entire database [19], primarily selecting countries for which the temporal COVID-19 evolution had reached peak intensity of the infection. Rather than including over 30 countries in the following discussion, we have chosen to focus on three groups, a total of 18 countries, that span the whole range of parameter values and could hopefully provide some insight to the multiple wave behavior of the pandemic. The choice of the 18 countries also aimed to represent parts of the world more heavily or less heavily impacted by the disease, as well as more typical cases. Here we defined the impact as the total number *N_T_* of infected individuals during the first 120 days of the pandemic, as predicted by the FSIR model [18]; this number is scaled by the population of the country (*N_P_*). In particular, we have included six countries in which the impact was small, China, Australia, Greece, Cyprus, Tunisia, and Japan for which (*N_T_/N_P_*)<1000 infected per million; six countries in which the impact was moderate, Israel, Denmark, Germany, France, Canada, and Portugal for which 1000<(*N_T_/N_P_*)<3000 infected per million; and six countries in which the impact was large, Sweden, Switzerland, United Kingdom, Italy, the United States, and Spain for which (*N_T_/N_P_*)>3000 infected per million.

We fit 7-day running averages of the daily data, for all countries, with data up to May 16, 2020. For each country, we estimated the number of waves (subepidemics) in which the infected population curve could be analyzed, the model parameters of each subepidemic, and the expected number of cases for the first major wave (N_T_^(1)^) and for all waves (*N_T_*). Table 1 presents the model parameters for the countries in our set.

**Table 1:** The values of the various parameters that enter in the multi-wave forced-susceptible-infectious-removed (FSIR) model of equation (6), for the representative countries considered.^a^

Code	Country	*t*_1_^(1)^ (days)	*N′* ^(1)^	*t*_1_^(2)^ (days)	*N′* ^(2)^	*t*_1_^(3)^ (days)	*N′* ^(3)^	*t*_1_^(4)^ (days)	*N′* ^(4)^	*N*_T_^(1)^	*N*_T_^b^
CHN	China	17.3	5868	N/A^c^	N/A	N/A	N/A	N/A	N/A	N/A	81,991
AUS	Australia	21.5	484	N/A	N/A	N/A	N/A	N/A	N/A	N/A	6770
GRC	Greece	6.21	44	21.5	112	44.5	39	N/A	N/A	2173	2707
CYP	Cyprus	9.0	49	30.7	11	N/A	N/A	N/A	N/A	677	821
TUN	Tunisia	9.7	50	21.7	23	N/A	N/A	N/A	N/A	686	1014
JPN	Japan	68.4	562	74.5	262	N/A	N/A	N/A	N/A	7870	14,966
ISR	Israel	21.6	799	40.3	377	N/A	N/A	N/A	N/A	11,179	16,453
DNK	Denmark	7.4	106	30.1	404	50.4	195	65.9	118	7076	11,467
FRA	France	26.8	5531	41.4	3309	62.2	1436	N/A	N/A	77,439	141,057
DEU	Germany	23.5	4937	33.5	4411	49.3	2328	69.1	1140	69,078	179,379
CHE	Switzerland	21.0	1368	34.4	615	52.5	186	N/A	N/A	19,128	30,365
PRT	Portugal	22.3	1066	39.6	716	60.9	381	N/A	N/A	14,911	30,281
CAN	Canada	30.6	1662	48.2	1921	63.3	1891	N/A	N/A	23,267	76,617
SWE	Sweden	12.6	143	32.4	650	50.5	776	69.3	754	11,081	32,476
ITA	Italy	22.9	6875	37.1	4062	51.5	3404	67.4	1583	96,243	222,918
GBR	United Kingdom	30.0	4744	43.6	5879	61.7	6630	N/A	N/A	66,414	241,489
ESP	Spain	25.1	10,389	40.5	3824	52.9	1639	74.1	1737	145,378	246,080
USA	United States	31.3	39,205	47.8	33,059	64.0	32,818	N/A	N/A	548,817	1,470,776

^a^The ordering of the countries is discussed in Table 2.

^b^The last column includes the values for the *expected* total number of cases *N_T_* when the number of infections has dropped to near zero and is an *extrapolated* value.

^c^N/A: Not applicable, as there is no relevant wave (subepidemic) for the respective parameter value to be obtained.

In the following, we present results obtained by the multiple-wave FSIR model for selected countries that can be accurately fitted by 4 waves (Italy, Sweden), 3 waves (United States, Portugal, Greece), and a single wave (China). The countries selected fall in two distinct classes: the first class comprises countries, which implemented stringent intervention measures rather fast; the second class comprises classes that implemented measures at rather later times and not at a high stringency level. The stringency of the measures is tracked daily by the Oxford COVID-19 Government Response Tracker (OxCGRT) [21], which systematically collects information on several different common policy responses governments have taken, scores the stringency of such measures, and aggregates these scores into a common Stringency Index. OxCGRT collects publicly available information on 17 indicators of government responses, that is, eight policy indicators recording information on containment and closure policies such as school closures and restrictions in movement, four indicators recording economic policies such as income support to citizens or provision of foreign aid, and five indicators recording health system policies. Italy, Portugal, Greece, and China had implemented high stringency measures rather fast, whereas Sweden, the United Kingdom, and the United States had not done so at that level.

**Figure 5:**
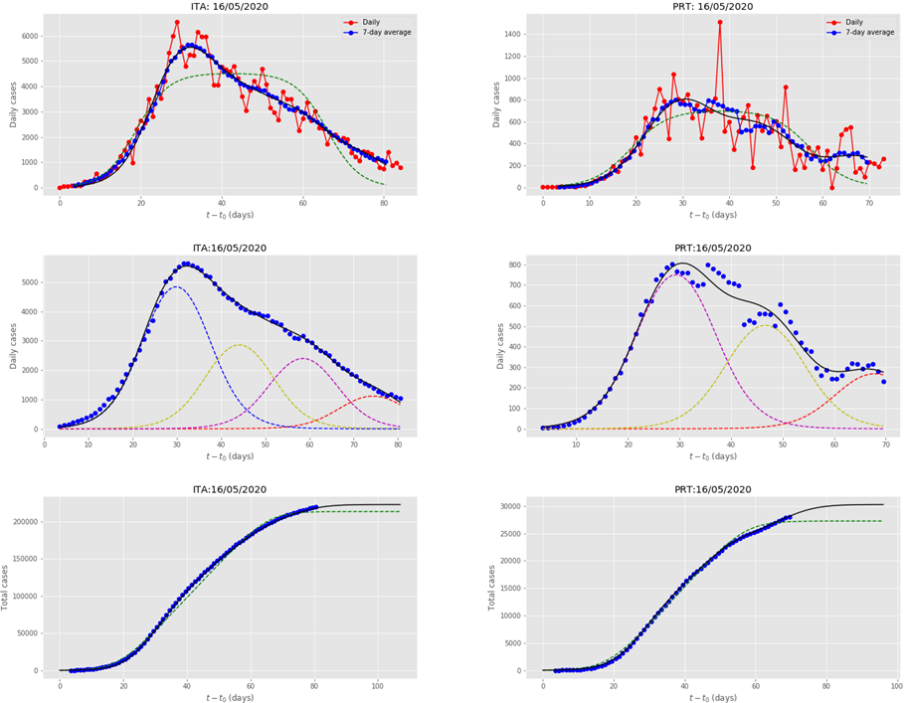
Results for ITA and PRT, obtained by fitting the multiple-wave forced-susceptible-infectious-removed (FSIR) model with data up to May 16, 2020. Top row: Red dots are the daily data reported by the European Centre for Disease Prevention and Control. The blue dots are 7-day running averages of the daily data. The green dashed line is the fit by the single-wave FSIR model. The black solid line is the 4-wave fit by the multiple-wave FSIR model. Middle row: Decomposition of the 7-day running average data (blue dots) in 4 waves for ITA and 3 waves for PRT. The black line represents the superposition of the multiple waves. The fit is in excellent agreement with the actual data. Bottom row: Blue dots are cumulative daily data (7-day running averages). The black line is the fit by the multiple-wave FSIR model, and it is essentially indistinguishable from the actual data. The green dashed line is the fit of the single-wave FSIR model, which clearly underfits the actual data. ITA: Italy; PRT: Portugal.

Figure 5 presents the multiple wave fit for Italy, which was one of the most heavily impacted countries by COVID-19. An initial large subepidemic was followed by 3 declining subepidemics. The use of the term “declining” (or its opposite, “increasing”) refers to the peak intensity of the subepidemic. Italy has taken strong intervention measures, since the country’s maximum stringency level was 94.29 [21]. The shape of the curve is reminiscent of the shape of the curve produced by the agent-based microscopic model, Figure 4 for *D*_0_=25. There is an excellent agreement between the 4-wave fit and the actual data in both daily and cumulative data. As can be seen, the single wave fit of the data, depicted by the green dashed lines, significantly underfits the data.

Figure 5 also presents the multiple wave fit for Portugal, which is a country experiencing a heavy impact by COVID-19. The country’s government has implemented stringent measures, with the highest stringency level at 89.52 [21]. The country’s incidence curve was fitted by 3 waves. An initial large subepidemic was followed by 2 declining subepidemics. Here too, the shape of the curve is reminiscent of the shape of the curve produced by the agent-based microscopic model, Figure 4 for *D*_0_=25. There is an excellent agreement between the 3-wave fit and the actual data in both daily and cumulative data. As can be seen, the 1-wave fit of the data significantly underfits the actual data. Italy and Portugal are representative examples of countries where the initial major wave is followed by several waves of *declining* strength, suggesting that, despite the initial large impact, the countries were successful in eventually containing the epidemic. Germany, France, Spain, Switzerland, Denmark, and Spain are exhibiting similar behavior, namely that of a major initial wave followed by several of declining strength.

**Figure 6:**
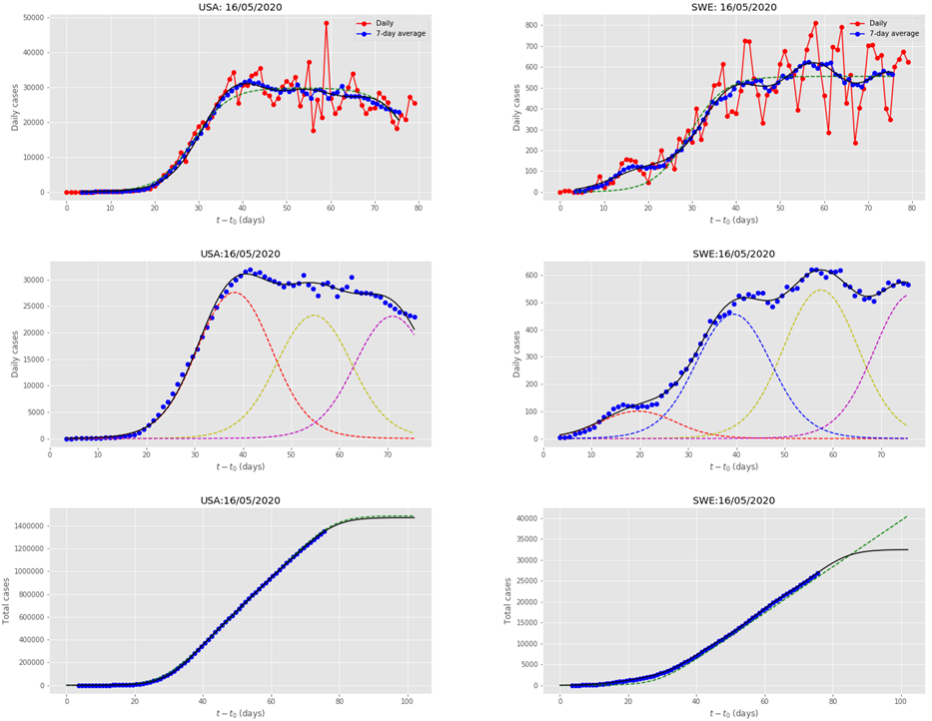
Results for the USA and SWE, obtained by fitting the multiple-wave forced-susceptible-infectious-removed model with data up to May 16, 2020. The meaning of symbols is the same as in Figure 5. SWE: Sweden; USA: United States.

Figure 6 presents the multiple wave fit for the United States, which appears to be the hardest hit country by COVID-19, in terms of total number of cases. The country implemented a series of intervention measures to stop the disease’s transmission and impact, which were deemed not to be taken aggressively enough, with highest stringency level 73.57 [21]. The country’s incidence curve was fitted by 3 waves. An initial large subepidemic was followed by subepidemics, with seemingly declining strength. However, the United States is a country comprising of more than 50 states and territories, and it is not clear if additional waves, possibly of strength comparable to the original ones, may materialize or not at later times. A study of decomposing the United States infected population curve per state is currently under way by the authors.

Figure 6 also presents the multiple wave fit for Sweden, which was also one of the hardest hit countries by the disease. However, the Swedish Government decided not to impose strict intervention measures but to inform the citizens to adopt certain precautionary measures, in a mostly individualistic capacity; the country’s maximum stringency level was 58.10 [21]. Thus, the country followed a different mitigation policy, with respect to the rest of Europe and most of the world. Sweden’s incidence curve was fitted by 4 waves. An initial small subepidemic was followed by 3 increasing subepidemics. It seems that the disease is spreading in waves; once a cluster of infected people is all removed, another bigger one is getting infected. Thus, the adoption of voluntary policy makes multiple nondeclining subepidemics of the disease get hold of the country. Since there was no clear trend as of May 16, 2020, of the country getting over the intensity peak, the 1-wave fit predicts a linear increase of the total number of expected cases. The 4-wave fit estimates a plateau of the total number of cases after the fourth wave, assuming that more waves do not materialize. According to the taxonomy of epidemic waves [13], COVID-19 in Sweden has generated an endemic wave; it remains to be determined if this is stationary or temporary.

Canada and the United Kingdom are among the countries exhibiting a similar subepidemics pattern, that of being impacted by a major wave followed by several waves of nondeclining strength.

**Figure 7:**
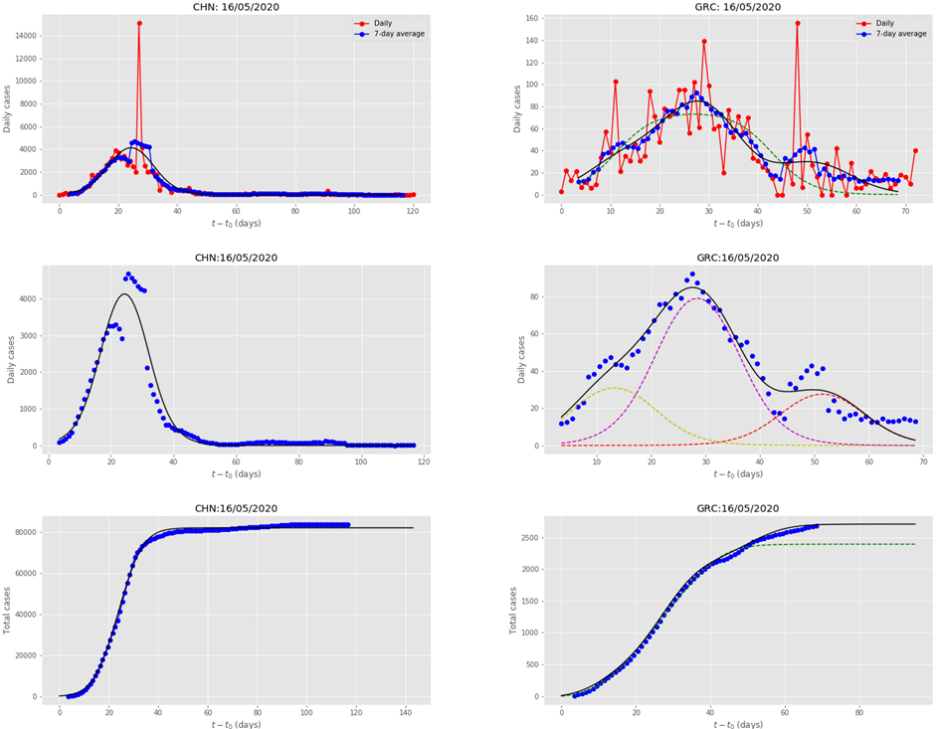
Results for CHN and GRC, obtained by fitting the multiple-wave forced-susceptible-infectious-removed (FSIR) model with data up to May 16, 2020. The meaning of symbols is the same as in Figure 5. CHN: China; GRC: Greece.

Figure 7 presents the single wave evolution of the disease in China, which was the first country to be hit by COVID-19, and the Chinese Government implemented a series of rather fast and strict intervention measures to stop the disease’s transmission and impact. The country’s incidence curve was fitted by a single wave.

Figure 7 also presents the multiple-wave fit for Greece, whose government quickly implemented a series of intervention measures to stop the disease’s transmission and impact, with highest stringency level 85.95 [21], keeping the total number of cases at very low levels. The country’s incidence curve was fitted by 3 waves. An initial small subepidemic was followed by a larger one, which was followed by one of declining strength, suggesting that it contained the epidemic efficiently and fast. Greece is a representative example of countries, such as Sweden and Denmark, which also exhibit an initial small wave followed by a larger one. However, Greece and Denmark countered the epidemic before reaching high levels of cases. In terms of declining subepidemics, Greece follows the pattern of countries such as Japan, Israel, Cyprus, and Tunisia, in which the initial major wave was followed by a single subepidemic of *declining* strength.

Similar to China, the imposition of strict measures in countries such as Australia and New Zealand, shows that countries were able to reduce the disease’s impact to a single wave. A quantitative estimate of the gain obtained is presented in the next-to-last column of Table 1, which presents the number of cases that could had been saved if countries had reduced the epidemic to a single wave rather than experiencing multiple ones; up to two-thirds of the total number of infections, as in the case of the United States, could have been avoided. Recent findings on the differential effects of intervention timing on COVID-19 spread in the United States [22] strongly corroborate this picture.

### The Pandemic Response Index

Countries respond to the pandemic in varied ways. It is an interesting question to quantify their varied response and make comparisons, which may be useful for contributing to the evaluation of the different policies followed. Based on the results of our model, it is possible to construct an index, the Pandemic Response Index (PRI), and assign a value to each country depending on its response to the pandemic.

To do this in an objective manner, we took into account two factors. First, the total number of infections as given by the quantity *N_T_* of Table 1, divided by the population of the country *N_P_*. The range of this quantity when multiplied by 100 is between 0 and 0.5 approximately. This is a measure of the overall impact of the pandemic on the population of the country, and as argued by our microscopic model, it is a reflection of how early measures to contain the epidemic were imposed. The second quantity we considered is ∆*N_T_*, the number of cases that correspond to all the waves except for the first major one, which in some cases includes the earliest small wave (see Table 2).

**Table 2:** Ranking of the various countries according to the PRI, defined in equation (7).

Code	Country	∆*N*_T_ (/million)^a^	*N*_T_ (/million)^b^	PRI^c^
CHN	China	0	59	9.94
AUS	Australia	0	271	9.73
GRC	Greece	50	252	8.76
CYP	Cyprus	121	690	8.44
TUN	Tunisia	28	88	8.32
JPN	Japan	56	118	7.51
ISR	Israel	594	1852	6.55
DNK	Denmark	757	1978	6.11
FRA	France	950	2106	5.64
DEU	Germany	1330	2163	4.76
CHE	Switzerland	1319	3565	4.59
PRT	Portugal	1495	2945	4.58
CAN	Canada	1440	2067	4.45
SWE	Sweden	2101	3189	3.52
ITA	Italy	2096	3689	3.47
GBR	United Kingdom	2633	3632	2.74
ESP	Spain	2155	5267	2.69
USA	United States	2818	4495	2.37

^a^∆*N_T_* is the difference between *N_T_* and the total number of cases that were infected by the first major subepidemic (for Sweden, Denmark, and Greece, both the small initial wave and the second wave have been taken into account). ∆*N_T_* has also been normalized by the country’s population in millions.

^b^*N_T_* is the asymptotic value after all waves have decayed, given in Table 1, normalized here by the country’s population in millions.

^c^PRI: Pandemic Response Index.

Arguably, this number of cases could have been avoided had the country imposed early and strict measures after the first wave of the epidemic was plainly evident; this was the case of single wave countries, for instance, China and Australia for which ∆*N_T_*=0. The larger this number is, the worse the performance of the country. This number, divided by 2*N_T_*, lies in the range 0-0.5. With these two quantities, we then define the “Pandemic Response Index” as:

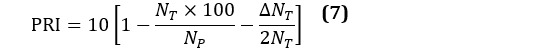



a quantity that lies in the range from 0-10, the higher values corresponding to better performance. This provides a quantitative and objective way of ranking the countries according to their performance. The results of this comparison and the relevant numbers that enter in the evaluation of the PRI are given in Table 2. We note that the classification is consistent with our initial selection of the countries considered here, as being those on which the disease had greater impact as measured by the number of infections per million, with arbitrarily chosen cutoffs in the ranges (smaller than 1000, between 1000 and 3000, and larger than 3000 per million). The only country that changes category based on the PRI value is Switzerland, which is raised to higher performance (average, see Table 2); this is a result of the fact that, although Switzerland had a relatively large number of cases per million (3565 cases per million), most of those occurred in the first wave, leaving a rather small percentage for subsequent waves. This remark suggests that the PRI is indeed a finer tool for evaluating performance, rather than relying on crude categorizations like the one based on the number of infections per million with arbitrary cutoff values between categories.

## Discussion

### Principal Findings

Reported cases of COVID-19 infections in various countries show features that are both common and regular, which we interpret as successive waves of transmission. We present evidence for this interpretation, using both agent-based simulations and a multi-wave model to fit the infected population data for many countries and give representative examples. This evidence supports the hypothesis that the COVID-19 pandemic can be successfully modeled as a series of epidemic waves (subepidemics). We analyzed the data from 18 countries based on this hypothesis and present the relevant parameters of a simple analytical model that accurately represents the data. Based on this analysis, it is possible to infer to what extent the imposition of early social distancing measures has slowed the spread of the disease. This analysis provides an estimate of how much lower the number of infections could have been, if early and strict intervention measures had been taken to stop the spread at the first wave, as actually happened for a handful of countries.

### Comparison With Prior Work

Recent works have emphasized more realistic approaches of human behavior and mobility involving larger transmission jumps by incorporating power-law decay of spatial interaction among human contacts [16], punctuated outbreaks as the disease progresses from one community to the next [12], and border effects [23]. In this study, our agent-based simulations start with 4 country-wide initial seeds for the disease onset in the “virtual country,” thus approximating in a reasonable way the effect of longer jumps before the imposition of the intervention measures such as social distancing and lockdown. Nevertheless, the microscopic model can be extended to encompass network and community structures as well as border effects by incorporating weighted interactions among the agents in the simulation grid.

The multiple-wave FSIR model can identify multiple waves (subepidemics), specifying *each one* by only three parameters, *t*_1_, ∆*t*, and *N*′, all of which are obtained by directly fitting the reported data of daily populations of infected individuals. Each of these parameters can be assigned a physical meaning, which help quantify certain generally held views; a detailed discussion of the meaning of these parameters can be found in [18]. Moreover, the quantitative picture that emerges from the values of these parameters produces a rather accurate picture of the severity of the epidemic in the various countries, and the effect of the intervention measures if and when any were taken.

A limitation of the original FSIR model is that it provides the extrapolation to future cases of infection as only a *lower limit*; this point has been discussed in an elegant mathematical analysis of the data by Fokas et al [17], highlighting the need for the inclusion of nonlinear terms in the underlying differential equations to capture the slow rate of the infected population decay. This is evident in the countries that have long passed the peak of the reported cases; the tail does not asymptote to a constant value, as the sigmoid (logistic) model predicts, but the number actually keeps growing at a slow rate. The multiple-wave FSIR mitigates this limitation of the original FSIR model; by modeling more accurately the wavy behavior of the infected population curve, it can provide a better fit to the daily data and to the cumulative actual data, and a better estimate to the cumulative number of cases (*N_T_*), as can be seen in all the cases we examined, see Figures 5 and 6.

### Limitations

The multiple-wave FSIR model may suffer from the fact that the number of infections dies off exponentially as the last wave does, a feature that appears unrealistic according to several other models that attempt to capture the long-term behavior [24-29]. Another limitation relates to the fact that in many cases, when ∆*t* is estimated as an adjustable parameter, it tends to provide an aggregate fit, that is, an initial large subepidemic tends to be followed by a longer in time and smaller in peak intensity averaged wave, which is the sum of smaller subepidemics. This wave can be characterized as a temporary endemic wave according to the taxonomy of [13]. To improve the resolution of the model and enable it to specify the underlying smaller subepidemics, an epidemiologically reasonable value for ∆*t* is necessary. Furthermore, caution must be exercised in interpreting the subepidemics because they may constitute a superposition of even smaller ones, as in the case of the United States, a country comprising 50 states with varied responses to the epidemic.

### Conclusions

Multiple waves of transmission during infectious disease epidemics represent a major public health challenge. Our agent-based simulations encompassing strong social distancing measures show epidemics with multiple wave structures. The analysis of reported data from 18 countries supports the hypothesis that the COVID-19 pandemic can be successfully modeled as a series of epidemic waves (subepidemics). The main strength of the simulations and the models developed and used in this work is the simplicity and the insight they offer on how the disease is transmitted in a country and on quantifying the effect of the intervention measures of the disease dynamics. Based on the model’s results, the construction of a PRI provides a finer tool for evaluating each country’s performance, instead of relying on crude categorizations like the one based on the number of infections per million with arbitrary cutoff values between categories.

## Abbreviations

COVID-19: coronavirus disease
FSIR: forced-susceptible-infectious-removed
OxCGRT: Oxford COVID-19 Government Response Tracker
PRI: Pandemic Response Index
SIR: susceptible-infectious-removed
